# Metabolomics variation profiling of vaginal discharge identifies potential targets for cervical cancer early warning: Vaginal discharge metabolomics potentially identifies cervical cancer

**DOI:** 10.3724/abbs.2022133

**Published:** 2022-09-16

**Authors:** Hanjie Xu, Min Liu, Yuexiao Song, Lou Liu, Feng Xu, Jiale Chen, Huiying Zhan, Ye Zhang, Yu Chen, Mudan Lu, Daozhen Chen

**Affiliations:** 1 The Affiliated Wuxi Maternity and Child Health Care Hospital of Nanjing Medical University Wuxi 214000 China; 2 Research Institute for Reproductive Health and Genetic Diseases the Affiliated Wuxi Maternity and Child Health Care Hospital of Nanjing Medical University Wuxi 214000 China; 3 Haidong No. 2 People’s Hospital Haidong 810699 China

Cervical cancer (CC) continues to be the second leading cause of cancer death in women aged 20 to 39 [1]. Recent research has shown that the cervical microbiota differs in different stages of cervical carcinogenesis [2]. It has been reported that the gut microbiota is linked to host metabolism, which influences the progression of the disease [3]. The role of cervicovaginal flora in affecting host metabolism, thereby causing the progression of cervical cancer, requires further research. In this direction, metabolomics has been implemented to detect small molecular differential metabolites in cancer progression. Metabolomics is the study of the thousands of low-molecular-weight molecules found in biological fluids and tissues of different individuals, whether normal or afflicted with disease, and it reflects reasonable changes in biological functions.

In this study, participants were consecutively recruited at the Affiliated Wuxi Maternity and Child Health Care Hospital of Nanjing Medical University. Forty nonpregnant women diagnosed with cervical dysplasia and CC, as well as healthy HPV-negative women, were enrolled in this study. This study was approved by the Institutional Review Boards of the Affiliated Wuxi Maternity and Child Health Care Hospital of Nanjing Medical University (Approval No: 2020-01-0309-06), and written informed consent was collected from each participant. The study has been registered in the Chinese Clinical Trials Registry (Registration No: ChiCTR2000034596, date: 2020/7/11). All cases were diagnosed by two professional doctors according to clinical and pathological features. Overall, patients were classified into 4 groups, i.e., women with low-grade squamous intraepithelial lesion (LSIL; *n*=10), women with high-grade squamous intraepithelial lesion (HSIL, *n*=10), women with CC ( *n*=10), and healthy participants ( *n*=10) as controls. The average age of the participants was 48.28 (SD: 12.76; range: 18‒78). The mean ages calculated for the HC, LSIL, HSIL and CC groups were 35.6, 54.7, 44.8 and 58.0 years, respectively. The clinical characteristics of the participants are presented in Supplementary Table S1.

The cervicovaginal metabolome signatures of each participant with or without cervical lesions were used to address the fundamental questions regarding the metabolic changes in cervical carcinogenesis. A representative LC-MS base peak chromatogram (BPC) of the identified metabolites is shown in Supplementary Figure S1, and the total ion chromatograms of all the samples are shown in Supplementary Figure S1. Although the overall trend of signals was approximately the same among the four groups, apparently specific signals were observed in each group. After peak alignment and removal of missing values, 16,927 electrospray ionization positive-mode (ESI+) features and 8672 negative-mode (ESI−) features were obtained. Further evaluation of these metabolomic profiles detected by XCMSonline revealed a total of 1907 out of 25599 features (1183 ESI+ and 724 ESI−), which exhibited significant differences among the 4 groups ( *P*<0.01; Supplementary Figure S2). The statistical evaluation by PCA showed separation among the four groups ( Figure 1A). The distribution of samples in PCA was resulted from the different levels of metabolic manifestations within the subjects and/or the date of subsequent sampling. To exclude possible confounding variables that were not related to the group differences and to evaluate the statistical significance of those signals, PLS-DA was applied ( Figure 1B). To explore potential biomarkers during the progression of cervical lesions, a pairwise comparison among patients was conducted with different statuses of cervical lesions. Finally, 270 features were selected based on the accumulation trend (either up or down) in at least one group from LSIL to HSIL to CC ( Figure 1E). Of the 270 differential metabolites, the 25 most significantly altered metabolites over the course of disease evaluated by the *P* and VIP values are shown in Figure 1F,G. Nucleotides such as NAD+, polyketides such as cohibin A and lipids such as glycerol triundecanoate showed a clear upwards trend in the CC group. Bacteriohopanepolyols, which are cell membrane lipids of prokaryotic organisms acting similarly to steroids in eukaryotic cells to enhance the stability of cell membranes, are increased in the CC group. Based on the 270 differential metabolites, PLS-DA was applied to sort out the components responsible for differences among the 4 groups. As shown in Figure 1C, the separation between groups is more clearly demonstrated in the counterpart of Figure 1B. From the PLS-DA plot, the overlap between the cervical lesion group and HC group was different, reflecting the possibility that cervical lesions can be warned by differential metabolites. Variables with a VIP score>1.0 were considered important contributors to the segregation ( Figure 1D), taking the HC group into account. Of note, among these metabolites, NAD+ was significantly elevated in CC patients, predicting the progression of cervical lesions ( Figure 1D,F,G). NAD+ exists widely in nature and participates in a variety of enzyme reactions. Previous reports showed that the level of NAD+ in HSIL patients is significantly reduced compared with that in HPV-negative women, and NAD+ is regulated by *Sneathia* [4], which may be significantly related to HPV infection, SIL and CC [5]. Our prediction of NAD+ is consistent with previous reports [4]. Together, our analysis indicated that metabolic disorders occur in the vaginal microenvironment of patients with cervical cancer, and certain metabolites can be considered biomarkers.

**Figure 1:**
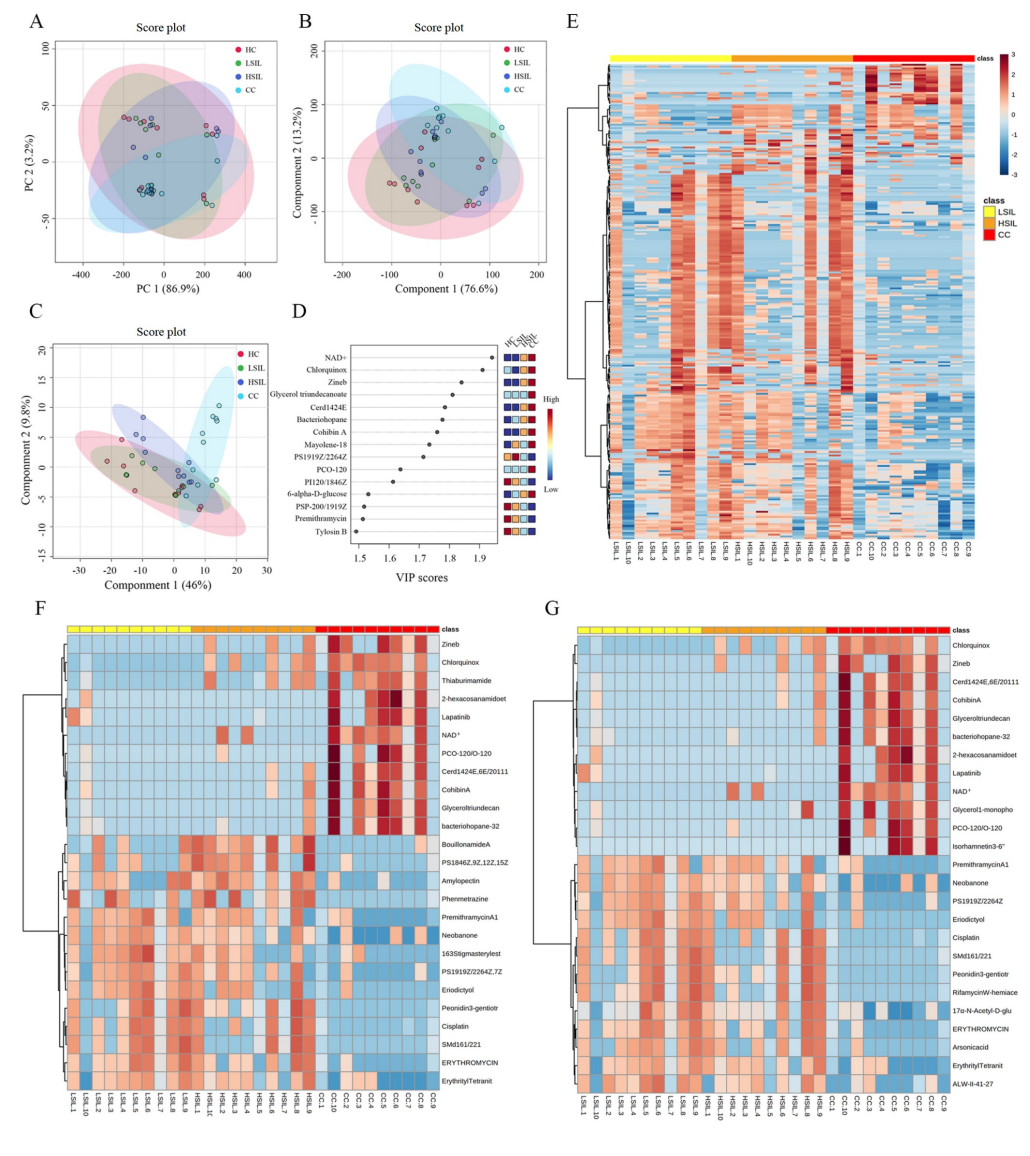
Figure 1 Changes in the metabolic profile and differential metabolites during the progression of cervical lesions Metabolic profiles of cervicovaginal samples by PCA (A) & PLS-DA (B), revealing the clustering of samples (R2X=0.98987, and Q2=0.12646). 2D score plot (R2X=0.6482, and Q2=0.40887) of 270 differential metabolites based on the PLS-DA model (C). Top 15 compounds based on VIP scores (D). Heatmaps of differential metabolites among cervical carcinogenesis. Study participant identification numbers are provided on the x-axis, and metabolites are listed on the y-axis. The heatmap depicted log-transformed relative intensity of the 270 differential metabolites (E), most variable metabolites valued by P value (F) and VIP value (G) between the LSIL, HSIL and CC groups.

To explore the metabolic pathways potentially responsible for the progression of cervical cancer, a KEGG pathway enrichment analysis was performed by using 270 differential metabolites and obtained 18 pathways ( Figure 2A and Table 1). Small *P* values and large pathway impart values indicated highly influenced pathways. Based on the *P* values and impart values, the pathway of phenylalanine, tyrosine and tryptophan biosynthesis (impact=0.8, *P*=0.08) was altered in the progression of cervical cancer, as shown in Figure 2B according to the KEGG pathway database ( https://www.kegg.jp/pathway/map00400#). Combining the results of KEGG pathway mapping, pathway enrichment analysis and relative concentrations of metabolites, L-tyrosine caught our attention ( Figure 2C). As reported, tyrosine, phenylalanine, and tryptophan have the potential to be biomarkers of gastroesophageal cancer [6]. Endogenous and exogenous amino acids are vital sources of nutrients distributed throughout the body to contribute to metabolism, gene expression, cell multiplication, and inflammatory reactions. The rapid proliferation and increased metabolism of tumor cells require amino acids for protein and nucleic acid synthesis [7]. The analysis of sera from cancer patients often shows altered amino acid profiles compared with healthy patients [8]. A larger group of amino acids in plasma, such as aspartate, glutamate, asparagine, and tyrosine, are gradually reduced from CIN to cervical cancer [9]. In another study, the concentration of tyrosine related to the citrate cycle was downregulated in blood samples collected from cervical cancer patients [10]. Consistent with previous research, decreased level of L-tyrosine in cervicovaginal discharge was detected in cervical cancer patients, compared with those in HSIL patients ( Figure 2). Thus, investigation of metabolic pathways associated with cervical cancer can give important insights into the different mechanisms adopted during the development and progression of tumors and provide new methods for early detection.

**Figure 2:**
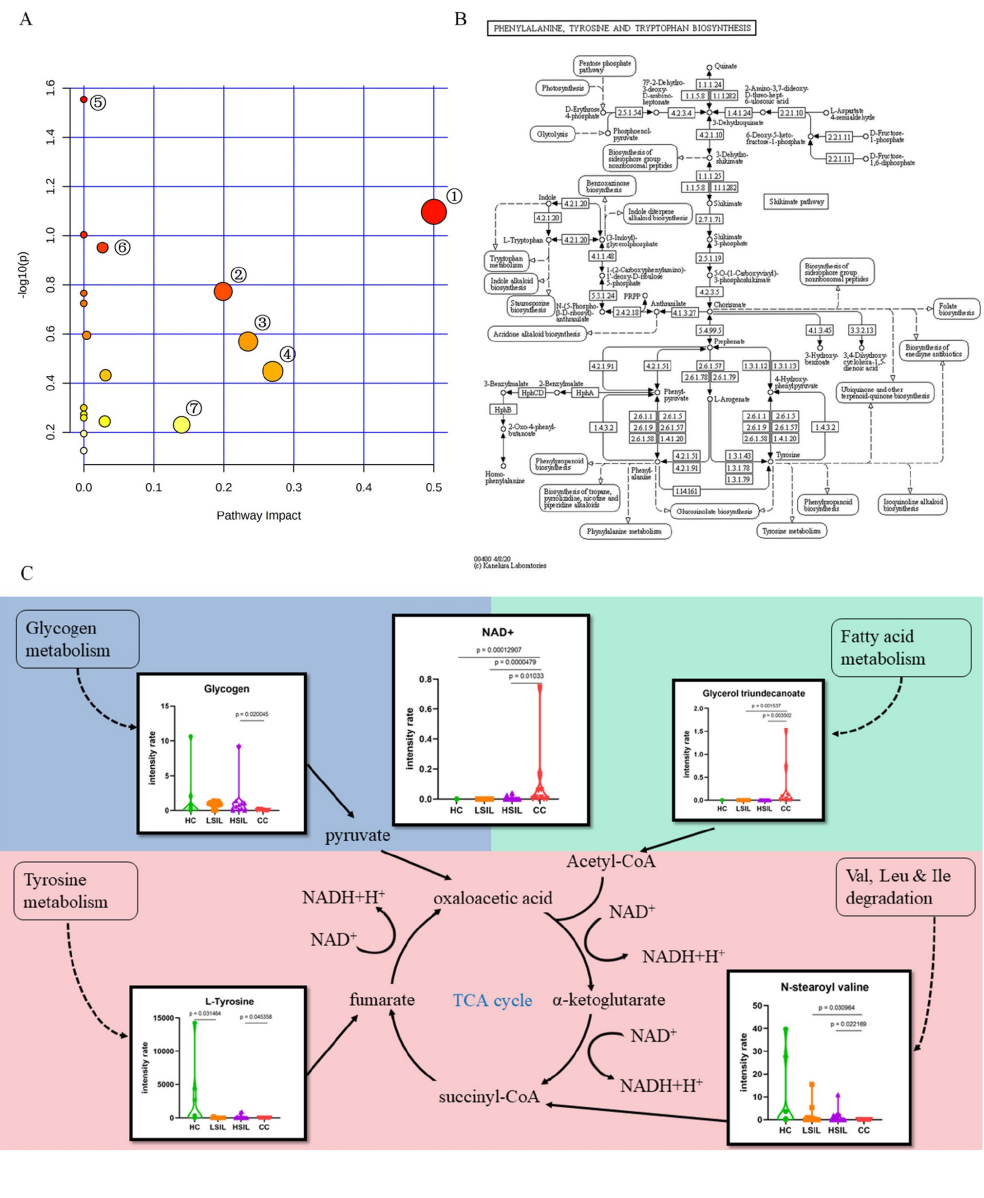
Figure 2 Changes in metabolic pathways during the progression of cervical lesions (A) Metabolic pathway analysis of cervical carcinogenesis. The 270 differential metabolites between HSIL and CC patients were used for pathway analysis conducted by Metaboanalyst: (1) Phenylalanine, tyrosine and tryptophan biosynthesis. (2) Glycerophospholipid metabolism. (3) Nicotinate and nicotinamide metabolism. (4) Sphingolipid metabolism. (5) alpha-Linolenic acid metabolism. (6) Glutathione metabolism. (7) Tyrosine metabolism. Model represents the tricarboxylic acid cycle metabolism pathway in cervical carcinoma. (B) Pathways of phenylalanine, tyrosine and tryptophan biosynthesis according to the KEGG pathway database (https://www.kegg.jp/pathway/map00400#). (C) Violin plots show the metabolites that were detected in our study. The state of the lesion is indicated on the x-axis of violin plots. The y-axis of violin plots represents the relative intensity of metabolites.

**Table:** **Table 1** Metabolite annotation and pathway enrichment analysis

Pathway name	Raw *P*	–Log ( *P*)	Holm *P*	FDR	Impact
Alpha-linolenic acid metabolism	0.027956	1.5535	1.0	1.0	0.0
Phenylalanine, tyrosine and tryptophan biosynthesis	0.080134	1.0962	1.0	1.0	0.5
Linoleic acid metabolism	0.099173	1.0036	1.0	1.0	0.0
Glutathione metabolism	0.11195	0.95097	1.0	1.0	0.02698
Glycerophospholipid metabolism	0.16881	0.77261	1.0	1.0	0.19895
Ubiquinone and other terpenoid-quinone biosynthesis	0.17159	0.76552	1.0	1.0	0.0
Phenylalanine metabolism	0.18879	0.72402	1.0	1.0	0.0
Glycosylphosphatidylinositol (GPI)-anchor biosynthesis	0.2542	0.59482	1.0	1.0	0.00399
Nicotinate and nicotinamide metabolism	0.26974	0.56905	1.0	1.0	0.23465
Sphingolipid metabolism	0.35659	0.44783	1.0	1.0	0.26978
Pyruvate metabolism	0.37005	0.43174	1.0	1.0	0.0311
Glycine, serine and threonine metabolism	0.50128	0.29992	1.0	1.0	0.0
Arachidonic acid metabolism	0.5322	0.27393	1.0	1.0	0.0
Arginine and proline metabolism	0.55177	0.25824	1.0	1.0	0.0
Valine, leucine and isoleucine degradation	0.57055	0.24371	1.0	1.0	0.02999
Tyrosine metabolism	0.58856	0.23021	1.0	1.0	0.13972
Aminoacyl-tRNA biosynthesis	0.63832	0.19496	1.0	1.0	0.0
Purine metabolism	0.7497	0.12511	1.0	1.0	0.0

The presented approach of a screening strategy for cervical cancer and precancer by cervicovaginal metabolomics is novel and promising. Nevertheless, large sample data and targeted metabolomics are needed to optimize and validate cervicovaginal biomarkers in further studies.

Taken together, cervical cancer is a serious but preventable global health problem. This study used metabolite analysis to test the hypothesis that the quantitative signatures of metabolites in cervical vaginal secretions can be used to describe the molecular changes of cervical cancer. This is a noninvasive approach that can be used to develop new strategies for the management of women at high risk of cervical cancer. Although more samples are needed to verify this approach, our data have preliminarily shown that it is a promising approach.

## Supplementary Data

Supplementary data is available at *Acta Biochimica et Biophysica Sinica* online.

## Supporting information

22160Supplementary_materials
