# Supplementary material for: Metabolomics variation profiling of vaginal discharge identifies potential targets for cervical cancer early warning: Vaginal discharge metabolomics potentially identifies cervical cancer
Source: Acta Biochim Biophys Sin (Shanghai). 2022 Sep 16;54(10):1561–5. doi: 10.3724/abbs.2022133 (PMC9827803; doi:10.3724/abbs.2022133)
Supplement: 22160Supplementary_materials [file 22160Supplementary_materials.pdf]

Supplementary Table S1. Clinical characteristics of the participants in each group

Participant details	HC	SIL		CC	*P*-value*
		LSIL	HSIL		
Age (mean, range)	35.6 (18–59)	54.7 (43–64)	44.8 (31–56)	58.0 (47–78)	0.000022*
Vaginal irritation, 24 hours prior					1.000000
Yes	0 (0.0)	0(0.0)	0 (0.0)	0 (0.0)	
No	10 (100.0)	10(100.0)	10 (100.0)	10 (100.0)	
Vaginal itching, 24 hours prior					1.000000
Yes	0 (0.0)	0(0.0)	0 (0.0)	0 (0.0)	
No	10 (100.0)	10(100.0)	10 (100.0)	10 (100.0)	
Vaginal discharge, 24 hours prior					1.000000
Yes	0 (0.0)	0(0.0)	0 (0.0)	0 (0.0)	
No	10 (100.0)	10(100.0)	10 (100.0)	10 (100.0)	
Vaginal burning, 24 hours prior					1.000000
Yes	0 (0.0)	0(0.0)	0 (0.0)	0 (0.0)	
No	10 (100.0)	10(100.0)	10 (100.0)	10 (100.0)	
Pain urinating, 24 hours prior					1.000000
Yes	0 (0.0)	0 (0.0)	0 (0.0)	0 (0.0)	
No	10 (100.0)	10 (100.0)	10 (100.0)	10 (100.0)	

Supplementary Table S2. List of exclusion criteria with verification methods

Exclusion criteria	Verification method
Any skin condition determined by the physician, which could interfere with the conduct of the study or increase the risks to the test subject including any open or healing cuts/ incisions, abrasions, or broken skin in the testing area as determined by the gynecologist	Self-reported and pelvic exam
Currently menstruating	Self-reported and pelvic exam
Currently using or have used antibiotics, antifungals, topical steroids or antiviral (oral or topical in the testing areas) within 7 days prior to visit	Self-reported and medical record
Current or history of genital herpes	Pelvic exam and medical record
Vaginal infection (including candidal infection or bacterial vaginosis), vulvar infection, urinary tract infection, sexually transmitted infection (chlamydia, gonorrhea, trichomoniasis, genital herpes) vaginal or urethral discharge (current or in the past 7 days)	Self-reported and pelvic exam
Using lotion, powder, ointment, cream, perfume or oil on the skin in the testing areas region 48 hours prior to visit	Self-reported
Using douching substances, vaginal medications, vaginal suppositories, and feminine deodorant spray, wipes, or lubricants to the genital area within 48 hours prior to the visit	Self-reported
Sexual intercourse within 48 hours prior to the visit	Self-reported
Current treatment for any skin conditions on the testing area	Self-reported
Have bathed or have gone swimming within 4 hours prior the visit	Self-reported
Have shaved, waxed or used depilatory treatments in the testing	Self-reported

area within 48 hours prior to the visit	
Type I or type II diabetes	Self-reported and medical record
Hepatitis or being HIV-positive	Medical record
Other medical conditions (such as an immunosuppressive condition), which in the investigator’s opinion would compromise their participation	Medical record

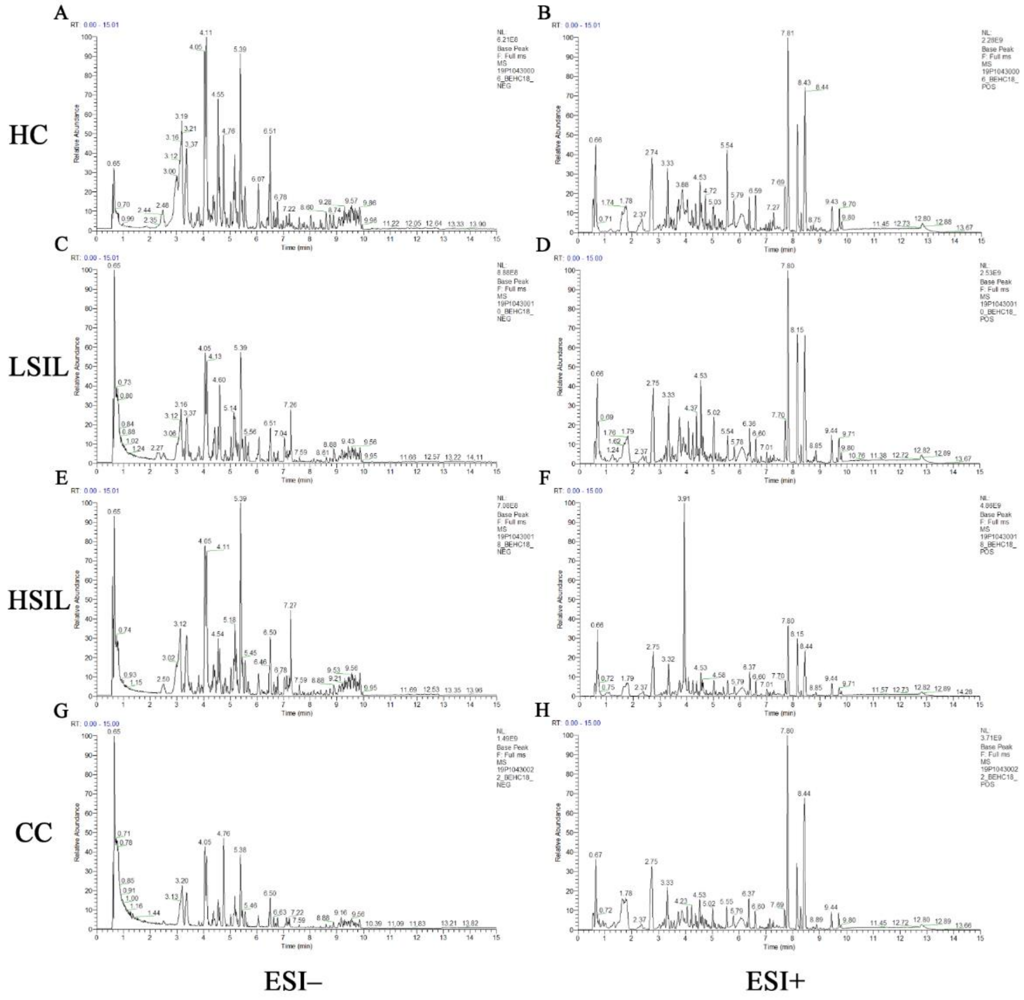

Supplementary Figure S1. Typical BPC from women with and without cervical lesions The typical base peak chromatogram (BPC) of cervicovaginal fluid in samples from four groups in ESI– (A,C,E,G) and ESI+ modes (B,D,F,H). The x-axis represents retention time, and the y-axis represents the charge-to-mass ratio of the features.

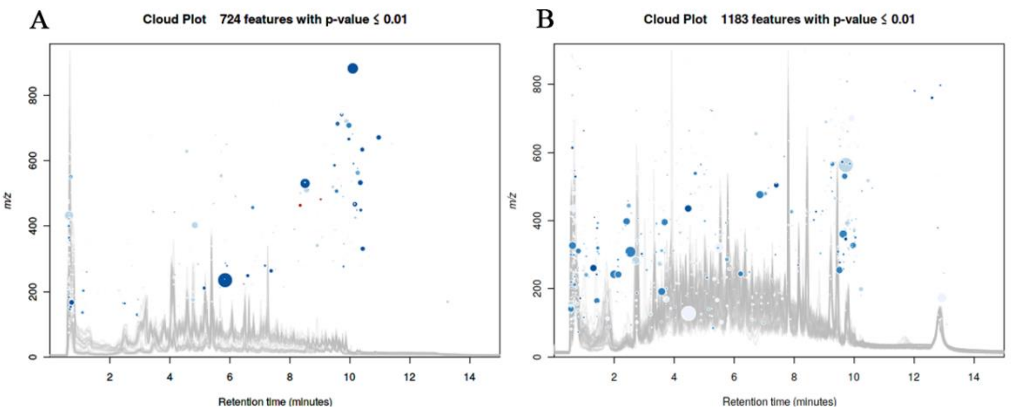

Supplementary Figure S2. Cloud plots coupled with total ion chromatograms of the ESI+ and ESI– modes After peak alignment and removal of missing values, 8672 electrospray ionization ESI− features (A) and 16927 ESI+ features (B) were obtained. The x-axis represents retention time, and the y-axis represents the charge-to-mass ratio of the features. Each circle in the cloud plot represents 1 differential feature, and the circle size represents the relative concentration of the feature.
